# Impact of Breastfeeding Barriers on Racial/Ethnic Disparities in Breastfeeding Outcomes in North Dakota

**DOI:** 10.1007/s40615-024-01943-z

**Published:** 2024-02-23

**Authors:** Anna Ross, Cora MacPherson, Lannesse Baker, Soojung Kim, Grace Njau, Andrew D. Williams

**Affiliations:** 1 Milken Institute School of Public Health, George Washington University, 950 New Hampshire Ave NW #2, Washington, DC 20037 USA; 2 University of North Dakota School of Medicine and Health Sciences, Room E166, 1301 North Columbia Road Stop 9037, Grand Forks, ND 58202-9037 USA; 3 Department of Communication, University of North Dakota, O’Kelly Hall Room 202, 221 Centennial Drive Stop 7169, Grand Forks, ND 58202-7169 USA; 4 North Dakota Department of Health & Human Services, 600 E Boulevard Ave Dept 325, Bismarck, ND 58505 USA

**Keywords:** Breastfeeding, Racial disparities, PRAMS, Indigenous, American Indian, Barriers

## Abstract

**Objective:**

Exclusive breastfeeding is recommended for the first 6 months of life, but there are racial/ethnic disparities in meeting this recommendation.

**Methods:**

2017–2020 North Dakota Pregnancy Risk Assessment Monitoring System (weighted *N* = 11,754) data were used to examine racial/ethnic differences in the association between self-reported breastfeeding barriers and breastfeeding duration. Breastfeeding duration was self-reported breastfeeding at 2 and 4 months, and number of weeks until breastfeeding cessation. Self-reported breastfeeding barriers were yes/no responses to 13 barriers (e.g., “difficulty latching,” “household duties”). Logistic regression estimated odds ratios and 95% confidence intervals to determine if barriers accounted for breastfeeding disparities by race/ethnicity. Cox proportional hazard models estimated hazard ratios for stopping breastfeeding for American Indian and other race/ethnicity individuals, compared to White individuals. Models were adjusted for birthing parents’ demographic and medical factors.

**Results:**

Logistic regression results suggest American Indian birthing parents had similar odds for breastfeeding duration (2-month duration: OR 0.94 (95%CI 0.50, 1.77); 4-month duration: OR 1.24 (95%CI 0.43, 3.62)) compared to White birthing parents, after accounting for breastfeeding barriers. Cox proportional hazard models suggest American Indian birthing parents had a lower hazard of stopping breastfeeding (HR 0.76 (95%CI 0.57, 0.99)) than White parents, after accounting for breastfeeding barriers.

**Conclusions:**

Accounting for breastfeeding barriers eliminated observed disparities in breastfeeding outcomes between American Indian and White birthing parents. Targeted and culturally safe efforts to reduce barriers to breastfeeding are warranted to reduce racial/ethnic disparities in breastfeeding.

**Supplementary Information:**

The online version contains supplementary material available at 10.1007/s40615-024-01943-z.

Breastfeeding has health benefits for both infants (improved cognitive development, reduced risk of asthma, obesity, and lower respiratory disease) [1] and breastfeeding parents (less likely to have postpartum emotional disturbances, breast cancer, ovarian cancer, and high blood pressure) [2]. Given the benefits of breastfeeding, Healthy People 2030 objectives include a target of 42.4% of infants exclusively breastfed at six months and a target of 54.1% of infants being breastfed with supplemental nutrition at 1 year [3].

Currently, only 1 in 4 infants is exclusively breastfed for the first 6 months of life in the United States (US) [4]. Breastfeeding rates differ among racial and ethnic groups, with the highest rate among the Asian/Pacific Islander population (83%), followed by White (78%), American Indian/Alaska Native (74%), and African American (60%) populations [5]. African American and American Indian/Alaskan Native individuals are also substantially less likely to be breastfeeding at 6 months and 12 months post-delivery compared with White individuals and Asian/Pacific Islander individuals [5]. In the US, the largest racial disparity in breastfeeding rates exists in North Dakota (ND), with a difference of 37.6 percentage points between the highest (Asian 91.6%) and lowest (American Indian/Alaska Native 54%) breastfeeding initiation rates [6]. As such, ND Maternal and Child Health Priorities include increasing the rates of breastfeeding initiation and exclusive breastfeeding through six months with an emphasis on the American Indian population [7].

Breastfeeding parents frequently cite several barriers to breastfeeding, such as practical obstacles like difficulty latching [5]. Brown and colleagues found that the most prevalent breastfeeding barriers among a Navajo community in Utah were practical breastfeeding difficulties like pain, difficulty latching, and milk supply, but additional information is needed to associate such barriers with breastfeeding outcomes in American Indian communities [8].

Other commonly reported barriers are external factors like work or school, and these barriers differ across racial/ethnic groups [5]. Certain barriers, like lack of social support and inadequate access to breastfeeding information, are more frequently reported by low-income racially minoritized individuals [5]. For example, an observational study focused on the south side of Chicago, a primarily African American community, reported patterns of breastfeeding rates and barriers in the area [9]. In primarily African American neighborhoods, rates of breastfeeding initiation were 1.4% and 6.8%, respectively, compared to 84.2% across the state of Illinois [9]. Low rates of breastfeeding were partially attributed to the lack of healthcare access in Chicago [8] and other low-income communities [10]. Low-income jobs do not typically have access to employer-sponsored Paid Family and Medical Leave programs. The unpaid federal Family and Medical Leave Act program is not financially attainable for many low-income families, and few states offer state-sponsored Paid Family and Medical Leave programs [11]. This economic burden [11] often leads individuals in low-income jobs to stop breastfeeding to return to work [5].

In American Indian communities, lack of access to resources is a barrier to positive health behaviors, but there are limited studies regarding breastfeeding outcomes. Concerning childhood obesity, American Indian parents reported that major barriers to the maintenance of a healthy weight include the cost of health-enhancing supplies, such as sports equipment and fresh produce, and a lack of safe spaces for children to engage in physical activity [12]. Furthermore, a sample of American Indian women indicated that limited availability of medical appointments and long wait times for copay reimbursements prevented them from seeking treatment for pelvic floor concerns [13], and the scarcity of available healthcare providers was shown to prevent American Indian mothers from receiving prenatal treatment [14].

Given the persistent disparities in breastfeeding in ND, an examination of barriers to breastfeeding that may contribute to disparate breastfeeding outcomes about American Indian women is warranted. In this study, we used the 2017–2020 ND Pregnancy Risk Assessment Monitoring System (ND PRAMS) data to examine the relationship between self-reported barriers to breastfeeding and time spent breastfeeding, with a specific interest in the disparity between American Indian breastfeeding parents and White breastfeeding parents. The purposes of the study are to provide data on self-reported barriers to breastfeeding, especially among American Indian individuals; to determine the association between self-reported barriers and breastfeeding duration; and to examine the role of self-reported breastfeeding barriers in the racial disparities in breastfeeding in North Dakota.

## Methods

This study used 2017–2020 (ND PRAMS data (https://www.hhs.nd.gov/prams). The ND PRAMS sample is randomly selected from all ND residents who delivered a live-born infant over the surveillance period. Selected birthing persons are contacted to participate 2–6 months after birth. Individuals from high risk, underrepresented groups are oversampled, and sample weights are applied to give unbiased estimates of population parameters after accounting for a stratified sample design, non-response, and noncoverage. Individuals are contacted by mail up to three times and by telephone once to complete a questionnaire collecting information on parental experiences before and after birth, health status, prenatal care, and substance use. The ND PRAMS 2017–2020 sample included 5788 respondents. Two thousand eight hundred eighty-seven individuals were excluded because they were missing data on breastfeeding initiation. Of those who responded, our analysis excluded individuals who did not report ever breastfeeding because these individuals were not prompted to respond about breastfeeding barriers. Our final sample included 969 individuals (weighted *N* = 11,754), all of whom had stopped breastfeeding at the time of completing the survey. Of the 4819 individuals excluded from this analysis, 51.1% were American Indian (compared to 8.4% of the overall sample), 77.5% were low-income (compared to 39.4% of the overall sample), and 43.9% had low education (compared to 30.2% of the overall sample). Of those who were still breastfeeding at the time of data collection, 21.9% were American Indian, 68.6% were white, and 9.5% were comprised of other racial and ethnic groups (compared to 49.3%, 7.8%, and 42.9%, respectively, for those who were no longer breastfeeding at the time of data collection). Additionally, of those who continued breastfeeding through the study period, 66.3% had an annual household income of at least $40,000 (compared to 35.9% of those who stopped), 13.4% were at least 35 years of age (compared to 11.4% of those who stopped), and 79.9% completed high school, compared to 57.9% of those who stopped (Online Resource 1).

### Breastfeeding Barriers

Self-reported barriers to breastfeeding were the primary independent variables of interest. Parents that had initiated breastfeeding, but had stopped at the time of the survey, responded to the question, “What were your reasons for stopping breastfeeding?” and possible barriers included: “My baby had difficulty latching or nursing,” “Breast milk alone did not satisfy my baby,” “I thought my baby was not gaining enough weight,” “My nipples were sore, cracked, or bleeding or it was too painful,” “I thought I was not producing enough milk, or my milk dried up,” “I had too many other household duties,” “I felt it was the right time to stop breastfeeding,” “I got sick or I had to stop for medical reasons,” “I went back to work,” “I went back to school,” “My partner did not support breastfeeding,” “My baby was jaundiced (yellowing of the skin or whites of the eyes),” and “Other.” Participants were allowed to select as many barriers to breastfeeding as applied. It is important to note that respondents were only prompted to answer this question if they had initiated breastfeeding in the past and stopped breastfeeding at the time of the survey.

### Race/Ethnicity

Self-reported maternal race/ethnicity information was collected. Categories include American Indian/Alaska Native (AIAN alone or biracial AIAN-White), White (White alone), and Other Racial Identities (other race/ethnicity; includes Black, Asian, Hispanic [all races], and other/unknown). Birth rates for the racial/ethnic groups captured by the Other Racial Identities category were not sufficiently large to complete analyses in terms of these groups. This categorization of maternal race/ethnicity aligns with PRAMS sampling methodology, and prior studies [15–18]. PRAMS sampling methodology is available online (https://www.cdc.gov/prams/methodology.htm) and in the ND PRAMS Sampling supplement (Online Resource 2).

### Breastfeeding Outcomes

The primary outcome of interest was time until breastfeeding cessation. Self-reported breastfeeding duration was provided via the question, “How many weeks or months did you breastfeed or feed pumped milk to your baby?” All responses were converted into weeks. We calculated three breastfeeding duration variables: binary variables (yes/no) indicating breastfeeding status at 2 months and 4 months after delivery; and the number of weeks (continuous) until participants reported stopping breastfeeding.

### Covariates

Covariates were selected based on existing literature [19]. The covariates of interest are parental age (younger than 35, and 35 and older); prenatal insurance type (Medicaid, other, and none), prenatal care adequacy (Kotelchuck index: Inadequate, Intermediate, Adequate, Adequate Plus), chronic illness (has at least one chronic illness, or no chronic illness), substance use (used tobacco or alcohol in the past 2 years, or did not use tobacco or alcohol in the past two years), weight classification (overweight or not overweight), postpartum depression (Always/Often, Sometimes, or Rarely/Never), history of depression (present, or not present), education (high school or more, or did not finish high school), income (less than $40,000 annually, or $40,000 or higher annually), use of Women Infants Children (WIC) program during pregnancy (yes, or no), adverse childhood experiences (Ace score: < 2, or ≥ 2), and pregnancy intention (later, sooner, at this time, not wanted, not sure), infant sleep position (back, non-back), and infant sleeping alone (always/often, sometimes/rarely/never). A description of all variables is included in Online Resource 3.

### Statistical Analysis

Statistical analyses were conducted using SAS 9.4. Frequencies of self-reported breastfeeding barriers were obtained for the overall sample and by maternal race. Logistic regression models were used to estimate odds ratios (OR) and 95% confidence intervals (95%CI) for the association between self-reported breastfeeding barrier, race, and each binary breastfeeding outcome (breastfeeding at 2 months and breastfeeding at 4 months). To examine breastfeeding barriers as the primary explanatory variables, we estimated crude associations between breastfeeding barriers and binary breastfeeding outcomes. Then, we added sociodemographic variables, health variables, prenatal care adequacy, and the use of WIC to generate adjusted odds ratios (OR). As the barriers to breastfeeding in PRAMS are heterogeneous, we first fit models individually for each barrier. Then, as participants were able to select all barriers that applied to them, we fit an additional model containing all barriers was fit to estimate the OR for each barrier after controlling for the others. To examine racial disparities, the same was used to generate crude and adjusted ORs comparing breastfeeding outcomes between racial groups. Survey weights were applied with the use of SAS Survey Procedures to account for a stratified sampling design in which groups of high interest were oversampled relative to the proportion they represent in the study population.

Additionally, cox proportional hazard models were used to estimate hazard ratios (HR) and 95% confidence intervals (95%CI) for time to cessation of breastfeeding among American Indian individuals, White individuals, and those from other racial groups. Self-reported weeks of breastfeeding duration were used for the hazard models. First, we estimated the crude hazard ratio for cessation of breastfeeding by race. Then, we adjusted for all the covariates and, finally, all the breastfeeding barriers.

As evidence suggests infant sleep may influence breastfeeding duration [20], we conducted sensitivity analyses by including infant sleep variables to the fully adjusted logistic regress models and Cox Proportional Hazard models.

This analysis was considered exempt by the University of North Dakota Institutional Review Board.

## Results

Respondents were primarily White, younger than 35 years of age, high-income, and had received at least a high school education (Table 1). American Indian individuals were more likely to report “household duties” and “school” as barriers to breastfeeding than White individuals (Table 2). Additionally, American Indian individuals reported “household duties,” “jaundice,” and “not gaining weight” significantly more often than individuals from other racial and ethnic groups.

**Table 1:** Sample proportions and 95% confidence intervals by race

	Total (weighted *N* = 39,302)	American Indian (weighted *N* = 3139)	White (weighted *N* = 31,037)	Other (weighted *N* = 5126)
Breastfeeding at two months	78.4% (0.78, 0.78)	60.2% (0.58, 0.62)	79.4% (0.79, 0.79)	80.7% (0.79, 0.81)
Breastfeeding at four months	69.3% (0.68, 0.70)	44.6% (0.42, 0.46)	71.1% (0.70, 0.71)	69.5% (0.68, 0.708)
Maternal age				
< 35	86.7% (0.86, 0.87)	90.6% (0.89, 0.91)	87.8% (0.87, 0.88)	79.9% (0.78, 0.81)
≥ 35	13.1% (0.12, 0.13)	9.38% (0.08, 0.10)	12.24% (0.11, 0.12)	20.13% (0.19, 0.21)
Income				
High	64.3% (0.63, 0.64)	19.6% (0.17, 0.21)	72.9% (0.72, 0.73)	33.0% (0.31, 0.34)
Low	35.7% (0.35, 0.36)	80.4% (0.78, 0.82)	27.1% (0.26, 0.27)	67.0% (0.65, 0.68)
Education				
More than high School	73.7% (0.73, 0.74)	50.5% (0.48, 0.52)	78.4% (0.77, 0.78)	56.3% (0.54, 0.57)
High school or less	26.3% (0.25, 0.26)	49.5% (0.47, 0.51)	21.6% (0.21, 0.22)	43.7% (0.42, 0.45)

**Table 2:**
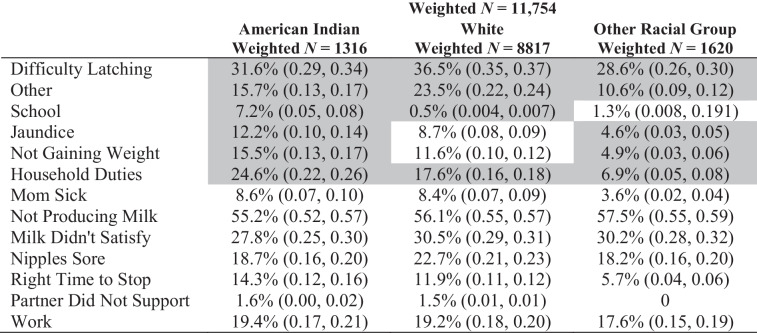
Distribution of “yes” responses by race and chi-squared tests for homogeneity of barriers

Shaded cells indicate statistically significant *χ*^2^ test (*p* < 0.05)

Logistic regression results for the association between breastfeeding barriers and breastfeeding outcomes are included in Table 3. Results suggest those who reported “difficulty latching” as a barrier to breastfeeding had 75% lower odds of breastfeeding at 2 months post-delivery (adjusted OR 0.25, 95%CI 0.12, 0.52) and 81% lower odds of breastfeeding at four months post-delivery (adjusted OR 0.19, 95%CI 0.06, 0.66), compared to those who did not report difficulty latching after adjusting for all covariates and other barriers. In the fully adjusted models, reporting “mom sick” or “work” as a barrier was associated with significantly higher odds of breastfeeding at 2 months (adjusted OR 4.85, 95%CI 1.27, 18.5; adjusted OR 4.70, 95%CI 2.13, 10.4), and reporting “right time to stop” was significantly associated with higher odds of breastfeeding at four months (adjusted OR 6.04, 95%CI 1.59, 23.0). Barriers associated with lower odds of breastfeeding at four months include “household duties” (adjusted OR 0.22, 95%CI 0.05, 0.93) and “not producing milk” (adjusted OR 0.39, 95%CI 0.16, 0.98).

**Table 3:**
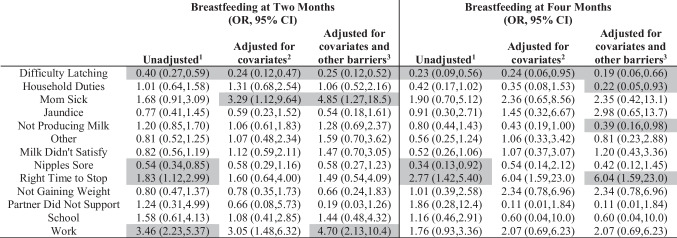
Odds ratios of breastfeeding outcomes by self-reported barriers

^1^Crude association between explanatory and outcome variable

^2^Adjusted for race, age, income, education, insurance used for prenatal care, use of WIC program during pregnancy, Kotelchuck index of prenatal care adequacy, ACE score, history of depression, history of chronic disease, substance use, overweight status, and postpartum depression

^3^Adjusted for covariates named above, plus all breastfeeding barriers

Shaded cells indicate statistically significant (*p* < 0.05)

Results for the odds of breastfeeding outcomes by race are included in Table 4. After adjusting for sociodemographic traits, medical history, prenatal care adequacy, and WIC use during pregnancy, American Indian individuals had 40% lower odds of breastfeeding at two months (adjusted OR 0.60, 95%CI 0.40, 0.90) and 46% lower odds of breastfeeding at four months (adjusted OR 0.54, 95%CI 0.37, 0.78), compared to White individuals. After adjusting for all the barriers to breastfeeding, there was no significant association between race and breastfeeding status at two months and four months after birth.

**Table 4:**
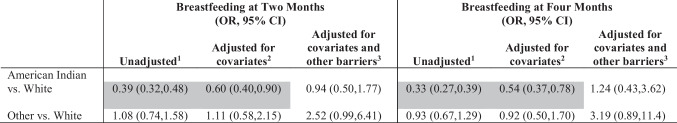
Odds ratio for breastfeeding outcomes by race

^1^Crude association between explanatory and outcome variable

^2^Adjusted for age, income, education, insurance used for prenatal care, use of WIC program during pregnancy, Kotelchuck index of prenatal care adequacy, ACE score, history of depression, history of chronic disease, substance use, overweight status, and postpartum depression

^3^Adjusted for covariates named above, plus all breastfeeding barriers

Shaded cells indicate statistically significant (*p* < 0.05)

**Fig. 1:**
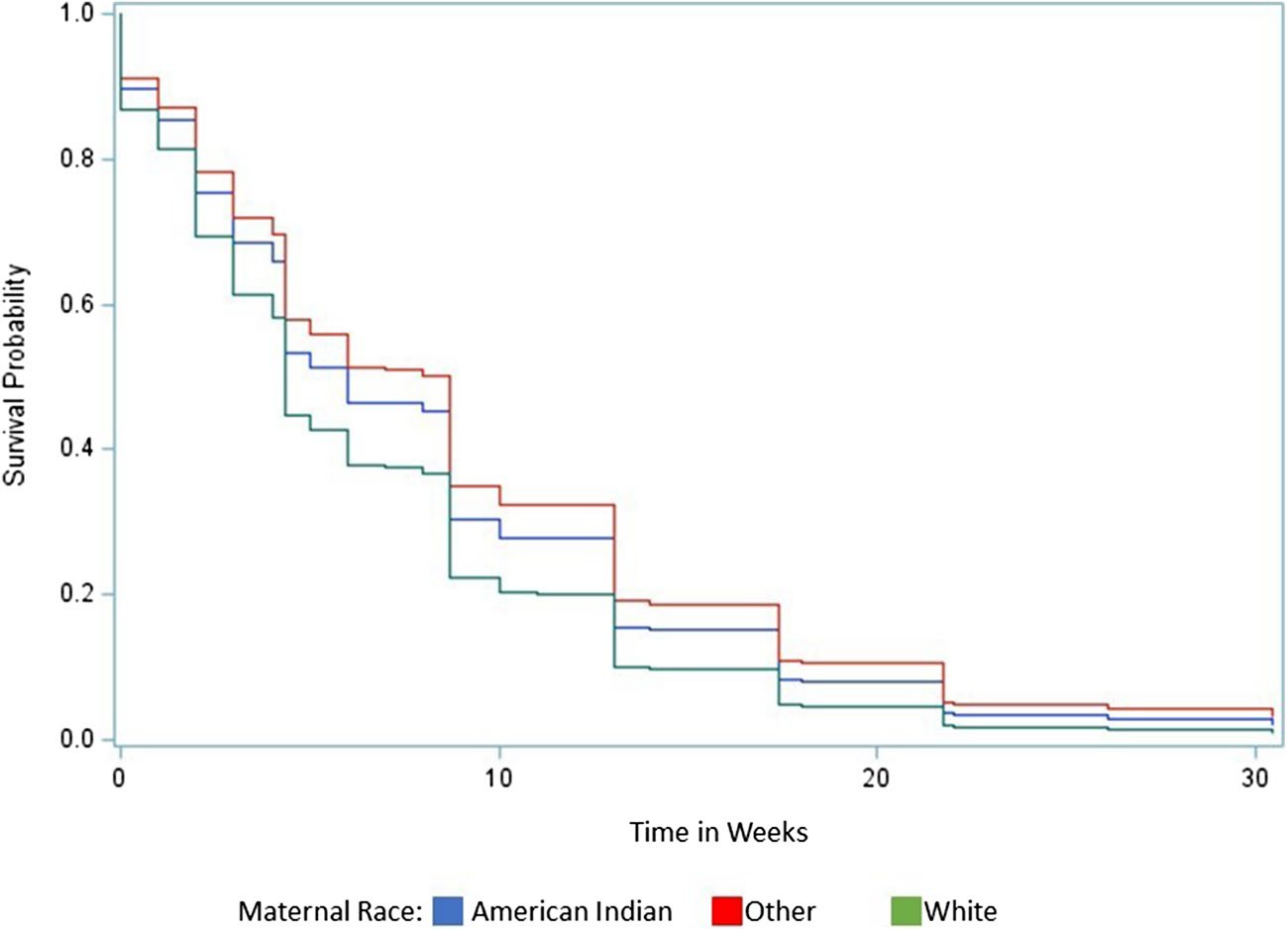
Fully adjusted survival curves for time to cessation of breastfeeding by race

Results for the survival analysis are included in Table 5 and Fig. 1. Only after adjusting for all breastfeeding barriers, American Indian individuals experienced 24% lower hazard of cessation of breastfeeding compared to White individuals (HR 0.76, 95%CI 0.57, 0.99). After adjusting for all self-reported breastfeeding barriers, the survival curves indicate that, at any given time over the entire time interval, Other race/ethnicity individuals have the highest survival probability (i.e., higher probability of continuing to breastfeed), followed by American Indian individuals and then White individuals.

**Table 5:** Hazard ratio for cessation of breastfeeding by race

	Hazard for cessation of breastfeeding HR (95% CI)		
	Unadjusted^1^	Adjusted for covariates^2^	Adjusted for covariates and other barriers^3^
American Indian vs. White	0.97 (0.85, 1.10)	0.83 (0.65, 1.04)	0.76 (0.57, 0.99)
Other vs. White	0.78 (0.63, 0.97)	0.73 (0.50, 1.06)	0.66 (0.44, 0.97)

^1^Crude association between explanatory and outcome variable

^2^Adjusted for age, income, education, insurance used for prenatal care, use of WIC program during pregnancy, Kotelchuck index of prenatal care adequacy, ACE score, history of depression, history of chronic disease, substance use, overweight status, and postpartum depression

^3^Adjusted for covariates named above, plus all breastfeeding barriers

In sensitivity analyses (Online Resources 4–6), the addition of infant sleep did not meaningfully change the observations from the fully adjusted models in Tables 4 and 5. For the hazard models (Online Resource 5), the confidence intervals for the American Indian group now includes 1.00 (HR 0.78, 95%CI 0.6, 1.02), yet the point estimate suggests lower risk of breastfeeding cessation compared to White individuals.

## Discussion

The purpose of this analysis was threefold: (1) report prevalence of barriers to breastfeeding, especially among American Indian individuals; (2) examine the relationship between self-reported barriers to breastfeeding and breastfeeding duration; and (3) examine if self-reported barriers to breastfeeding contribute to racial disparities in breastfeeding in ND.

Our observation of shorter breastfeeding duration among American Indian women compared to other racial/ethnic groups aligns with breastfeeding duration from national PRAMS data [21] and lower breastfeeding initiation among American Indian women in multistate birth certificate data [22]. Overall and for each racial/ethnic group, the most frequently reported barriers to breastfeeding were not producing milk, difficulty latching, and milk did not satisfy. This is consistent with a cross-sectional study examining breastfeeding behavior in a rural Navajo community that found practical breastfeeding difficulties like pain, latching concerns, and milk supply were associated with stopping breastfeeding prior to 6 months after delivery [8]. Notably, Navajo community members expressed interest in lactation support, which may have alleviated some of these difficulties, but reported not having access [8].

Additionally, American Indian respondents reported household duties and school as barriers to breastfeeding significantly more often than White respondents. These results can be linked to previous findings, which have shown breastfeeding behavior to be influenced by community, culture, and family in American Indian populations. An ethnographic study of Ojibwe communities in Minnesota identified mixed messages regarding benefits of breastfeeding, life circumstances, nurturing and support, and traditions as the four main patterns of cultural influence on breastfeeding behavior [23]. Their observation of housework as a “life circumstance” impacting breastfeeding outcomes and its relationship with economic class is reflected by our finding that household duties are associated with the cessation of breastfeeding. Furthermore, Indigenous birthing people frequently consult family members and elders in the community before making the decision to breastfeed or not [23]. Brown and colleagues found that babies’ fathers significantly influenced breastfeeding behavior in the Navajo community, reporting that those who breastfed for less than six months were often affected by the non-breastfeeding parent [8]. Interventions intended to increase breastfeeding behavior in American Indian birthing people must account for the social and cultural norms that are specific to this community. Existing evidence suggests that the strength of cultural traditions related to breastfeeding and the involvement of female family members are associated with infant feeding behavior [24, 25]. Ojibwe traditions were observed to be highly supportive of breastfeeding parents, and Ojibwe respondents expressed a distrust of Western medicine and a preference for Indigenous health practitioners [23]. Programs targeting American Indian breastfeeding parents should prioritize culturally competent medical providers and the utilization of traditional approaches rather than relying on Western medicine alone. Further research should examine the unique qualities of home life and family dynamics (e.g., cultural education, familial and community support, father support, supportive communication about breastfeeding) in American Indian communities that may contribute to environments more conducive to the continuation of breastfeeding, which can inform culturally relevant programs for breastfeeding promotion.

The present study provides evidence that the disparity in breastfeeding rates between American Indian and White individuals may be a consequence of breastfeeding barriers. The unadjusted logistic regression models show that American Indian individuals are significantly less likely to continue breastfeeding at 2 months and 4 months after delivery, but adjusting for all the breastfeeding barriers mentioned in the ND PRAMS survey reduced this difference to the point of no significance. In other words, accounting for self-reported breastfeeding barriers (i.e., household duties, school) improved outcomes for American Indian birthing people to an extent that the disparity with the White group was eliminated. This would suggest that the difference in breastfeeding rates between American Indian and White individuals could be attributed to the breastfeeding barriers and eliminating such barriers could contribute to mitigating the persistent disparity.

Furthermore, adjusting for breastfeeding barriers in the hazard models reduced the risk of cessation of breastfeeding by 21% for American Indian individuals compared to White respondents. The directionality of this effect suggests that adjusting for self-reported barriers to breastfeeding substantially improves breastfeeding duration for American Indian birthing people. It is worth noting that the lack of a significant association between race and time to cessation of breastfeeding in the unadjusted Cox PH models is inconsistent with the report from Jones and colleagues that American Indian and Alaska Native individuals have significantly higher rates of cessation of breastfeeding than the general US population [5]. Possible reasons for this discrepancy may be related to characteristics unique to our sample, which only includes individuals who initiated breastfeeding, stopped breastfeeding before the survey follow-up, and answered the question about breastfeeding barriers. Those excluded from our analysis were 51.1% American Indian, so our results are likely biased toward White individuals. The directionality of this effect suggests that adjusting for self-reported barriers to breastfeeding significantly improves breastfeeding duration for American Indian birthing people.

To address the observed disparity, consideration should be given to the breastfeeding barriers with the greatest impact on breastfeeding outcomes. Among the sample, overall and for each racial/ethnic group, the most frequently reported barriers to breastfeeding were not producing milk, difficulty latching, and milk did not satisfy. In our analyses, difficulty latching was associated with lower odds of continuing breastfeeding at 2 months and 4 months, and not producing milk was associated with lower odds of breastfeeding at 4 months (Table 3). A systematic review of breastfeeding interventions provided evidence that lactation consultants and counselors improve breastfeeding outcomes [26]. However, a lack of access to health resources is a major factor in the health behaviors among American Indian communities [12–14]. Specific to breastfeeding, American Indian populations in the US [8], and communities across ND have limited access to lactation support [27]. For example, there were 2.26 International Board Certified Lactation Consultants (IBCLCs) per 1000 live births in ND in 2014, compared to an average of 3.48 in the United States overall [27].

The time of impact of each breastfeeding barrier may also provide insight into when a particular intervention is most appropriate. In our sample, those who reported difficulty latching and not producing milk typically stopped breastfeeding by the 4-month point. These results suggest that, in the earlier postpartum months, practical difficulties of breastfeeding prevent birthing parents from maintaining breastfeeding behavior. This is consistent with previous findings that, in communities with low rates of breastfeeding initiation, there is typically a severe lack of access to lactation professionals that could help breastfeeding individuals overcome these obstacles [28]. We build on this data by demonstrating that inadequate lactation support is detrimental to not only initiation but also continuation of breastfeeding. To be most impactful, lactation support should be prioritized during the initial phase of the postpartum period.

Currently, the ND Department of Health & Human Services is partnering with the Indian Health Service and tribal communities to implement a doula pilot project in a tribal community [29]. This doula project may directly address the practical difficulties of breastfeeding, such as difficulty latching, via improved access to breastfeeding education. Furthermore, breastfeeding initiation rates increased faster among American Indian women enrolled in WIC than WIC-eligible American Indian women not enrolled in WIC, suggesting culturally safe approaches to enroll eligible American Indian women in WIC may be successful to improve breastfeeding outcomes [22]. Initiatives such as tribally focused doulas and culturally safe WIC programs should be evaluated to provide evidence to inform future efforts to improve breastfeeding rates among American Indian parents.

On the other hand, those who reported illness or work as a barrier had significantly higher odds of breastfeeding at 2 months, but no such difference existed at the 4-month mark. In other words, for those who stopped breastfeeding due to illness or work, their stopping point was typically after 2 months. This would imply that individuals who were able to overcome the initial hurdles of breastfeeding difficulties were frequently forced to stop breastfeeding upon returning to work sometime after 2 months post-delivery. Previous data report that workplace-specific breastfeeding barriers include inadequate time, breastfeeding pump access, and pumping facilities, as well as unsupportive company cultures around pumping [28]. Furthermore, expansion of paid Family and Medical Leave may reduce economic hardship facing low-income racially minoritized families—including American Indian families—compared to these families relying on unpaid leave for medical circumstances [11]. Thus, the expansion of paid Family and Medical Leave to workers in low-income jobs may help address economic and workforce barriers to longer breastfeeding duration. Encouraging breastfeeding-positive workplace policies is an important step to support the goal of continued breastfeeding for the entire first year.

This study has several limitations. Self-reported survey data lends itself to non-response, social desirability, and recall biases. Individuals only provided responses about barriers to breastfeeding if they stopped breastfeeding during the study period. As such, the survey data does not provide information about the barriers faced by individuals who continued breastfeeding beyond their participation in the survey. This information, if collected, may have allowed insight into which breastfeeding barriers were surmountable. Those who were excluded due to non-response of the barriers question had a higher proportion of American Indian, low-income, and low-education individuals than the overall sample. As such, these results are likely underestimating barriers for these groups. However, ND PRAMS data is collected 2 to 6 months after delivery, and the self-report measures used in PRAMS have demonstrated high validity and reliability overall [30]. Of additional concern is that PRAMS is collected via mail and phone interview, and individuals experiencing housing instability or inadequate phone access—which disproportionately effect American Indian people—may be underrepresented [31–33]. American Indian individuals represented 30% of ND’s homeless population, and only 40% of American Indian residents own their homes [34]. Additionally, this study offers limited information on racial groups besides American Indian and White due to insufficient sample size. Therefore, the results of this study cannot be generalized to racial and ethnic groups besides these two. However, by providing some of the first data on breastfeeding barriers among Northern Plains American Indian individuals, these findings are a valuable step in efforts to reduce the wide disparity of breastfeeding behavior in ND.

In conclusion, different breastfeeding cessation rates between American Indian and White birthing people in ND appear to be attributable to the presence of breastfeeding barriers. Research associating barriers with breastfeeding outcomes in American Indian individuals is limited, and this project highlights the importance of investigating such barriers. To best inform efforts to mitigate racial/ethnic disparities in breastfeeding outcomes, additional research using varied methods of inquiry is required, most notably examinations within the diverse tribal populations that encompass the broader American Indian group. Policies and programs specifically aimed at overcoming barriers to breastfeeding, while promoting cultural safety and humility, are necessary to address racial/ethnic disparities in breastfeeding.

## Supplementary Information

Below is the link to the electronic supplementary material.

Supplementary file1 (DOCX 20 KB)

Supplementary file2 (DOCX 57 KB)

Supplementary file3 (DOCX 18 KB)

Supplementary file4 (DOCX 18 KB)

Supplementary file5 (DOCX 17 KB)

Supplementary file6 (DOCX 55 KB)

## Data Availability

North Dakota PRAMS data are available after project approval from the North Dakota Department of Health and Human Services: https://www.hhs.nd.gov/prams.
