# Supplementary material for: Impact of Breastfeeding Barriers on Racial/Ethnic Disparities in Breastfeeding Outcomes in North Dakota
Source: J Racial Ethn Health Disparities. 2024 Feb 23;12(2):1063–72. doi: 10.1007/s40615-024-01943-z (PMC11913940; doi:10.1007/s40615-024-01943-z)
Supplement: Supplementary file 1 — Supplementary file1 (DOCX 20 KB) [file 40615_2024_1943_MOESM1_ESM.docx]

**Online Resource 1. Description of excluded sample by key demographic variables and breastfeeding status**

|  | **Continued Breastfeeding** | **Stopped Breastfeeding** |
| --- | --- | --- |
| **Race/Ethnicity** |  |  |
| American Indian | 21.9% | 49.3% |
| White | 68.6% | 7.8% |
| Other | 9.5% | 42.9% |
| **Maternal Age** |  |  |
| <35 | 86.6% | 88.6% |
| ≥35 | 13.4% | 11.4% |
| **Income** |  |  |
| High | 66.3% | 35.9% |
| Low | 33.7% | 64.1% |
| **Education** |  |  |
| More than High School | 79.9% | 57.9% |
| High School or less | 20.1% | 42.1% |
