# Supplementary material for: Impact of Breastfeeding Barriers on Racial/Ethnic Disparities in Breastfeeding Outcomes in North Dakota
Source: J Racial Ethn Health Disparities. 2024 Feb 23;12(2):1063–72. doi: 10.1007/s40615-024-01943-z (PMC11913940; doi:10.1007/s40615-024-01943-z)
Supplement: Supplementary file 2 — Supplementary file2 (DOCX 57 KB) [file 40615_2024_1943_MOESM2_ESM.docx]

# Online Resource 2. ND PRAMS Sampling Description

# 4 Sampling

## Definition

The population of interest for PRAMS is all mothers who are residents of North Dakota who delivered within North Dakota a live-born infant during the surveillance period. To draw a sample from this population, a *sampling frame* must be identified. The sampling frame is a list of mothers that represents the population eligible for inclusion in the sample. Often a complete list of all resident women who delivered a live-born infant within the state does not exist. Therefore, the operational sampling unit for PRAMS is *infants* who were born alive within North Dakota to resident mothers during a specified interval. North Dakota's vital records birth certificate file serves as the best available source of the sampling frame representing live births.

By definition, the target population for PRAMS is limited to pregnancies resulting in a live-born infant. By using the birth certificate file as the sampling frame, PRAMS implicitly excludes stillbirths, fetal deaths and induced abortions. The reason for this is twofold: 1) reporting systems for these outcomes are not routinely in place in many states, and 2) the standard definitions for these outcomes vary widely.

Because of the importance of learning about the maternal behaviors of mothers whose infants have died, they will be *included* in the sampling frame. The original PRAMS states were concerned that the inclusion of sampled mothers whose infants had died or had severe health problems would affect response rates because of a possible lack of sensitivity in approaching them. PRAMS data analysis has found that the response rates for mothers of infants who have died are comparable or higher than the response rates for other mothers. The literature states, and PRAMS experience indicates, that these mothers are usually eager to participate in epidemiologic studies because they want to help researchers understand the problems that their babies experienced. PRAMS uses a separate letter for these women that expresses sympathy for their loss and encourages their participation by emphasizing that their input may be helpful to the health of other mothers and babies. Additionally, the questionnaire is sensitive to this issue and has had little difficulty in eliciting responses from this group of women.

## Adjustments to the Sampling Frame

***4.2a Exclusions.*** Exclusions are made because of particular concerns or operational difficulties in including them in the sample. These exclusions are described here.

1. ***Out-of-State Births to Residents.*** For practical reasons, the sampling frame is restricted to infants who were delivered in North Dakota. There are often substantial delays in obtaining birth certificate information from other states. In most cases these records are obtained too late to be sampled and followed up within the two to six month time frame prescribed in this protocol.
2. ***In-State Births to Nonresidents.*** As in-state births to nonresident mothers may be difficult to trace, they will be excluded from the sampling frame. Furthermore, a state's target population for public health action does not extend beyond its residents, and information on residents would be more relevant for better serving the public health needs of North Dakota.
3. ***Missing Information.*** Infants whose birth certificates lack the mother's last name are excluded from the sampling frame because this information is crucial for follow-up. Birth certificates that are only missing a mailing address will *not* be excluded because often it is possible to obtain that information from another source. Birth certificates missing any other information will not be excluded.
4. ***Delayed or Early Processing of Birth Certificates.*** Birth certificates that are processed too late after the birth occurred (more than six months afterwards) are excluded from the sampling frame. Use of these records would raise concerns about recall bias, ability to locate the mother, and comparability with other respondents. Birth certificates that appear on the sampling frame less than two months after the birth occurred will be temporarily excluded. These records will be included in the sampling frame two months after the birth.

### Protocol Development Task

Estimate to what extent delayed receipt of information and/or early processing of birth certificates will affect your state’s sampling procedures in the format below. In the following table, this information is translated into estimates of the percentage of births in a given month that will be available for inclusion in the sampling frame by the specified number of days after birth.

Births Available on Computer File for Sampling

| Number of Days After Birth | 30 | 60 | 90 | 120 | 150 | 180 | 210 | 240 |
| --- | --- | --- | --- | --- | --- | --- | --- | --- |
| Percent of Births on File | 98.2 | >99 | >99 | >99 | >99 | >99 | >99 | >99 |

1. ***Multiple Gestation Infants.*** The issue of multiple gestation in the sampling frame is complex. These infants are of interest because of the elevated rates of low birth weight and infant mortality. However, infants of a multiple gestation have had the same intrauterine environment, thus are not independent of one another. It is necessary to establish measures to ensure that only one infant of a multiple gestation is included in the sampling frame.
   - For each twin or triplet set, *one member* will be randomly selected for inclusion in the sampling frame. If one of the deliveries results in a fetal death, the surviving twin or triplet(s) will be included. **See** **Section 4.4a** for the procedure used to make this selection.
   - Multiple gestations involving four or more siblings, however, should be excluded from the sampling frame. Because of the rarity of multiple births of this number, little is lost in representing the population. Quadruplets and quintuplets are almost always below normal birthweight, but not because of risk factors that are of interest in epidemiologic studies. Alerted to possible complications, the mother most likely received more than the usual prenatal care. Because of the publicity that often surrounds such births, the mother's pregnancy is unlikely to have been representative of the general experience.
2. ***Adopted Infants.*** The sampling of adopted infants can also be a sensitive issue. The adoptive mother is often named on the birth certificate. As the majority of the survey questions involve the time period prior to and during the pregnancy, the adoptive mother is not qualified to respond to the PRAMS survey. For this reason, any adoptions that are identified as such on the birth certificate should be excluded from the sample.

It is not always possible to identify adoptions before the sample is drawn. If an adoption is not identified for exclusion prior to drawing the sample but is discovered during the data collection period, one of two situations may occur. In the first case, if the name of the adoptive mother is provided on the birth certificate, this mother should be dropped from further follow-up for the reasons given above (using the “Drop Mom” feature in the PRAMS software system). If, however, the name of the birth mother is provided on the birth certificate, PRAMS staff should attempt to contact the mother and encourage her to participate. This mother is able to answer the questions regarding her experiences prior to and during the pregnancy. The survey has been designed to allow these mothers, whose infants do not live with them, to skip the questions pertaining to the child’s early infancy.

### Protocol Development Task

Explain how adoptions are processed in your state and explain if and how they are identifiable for exclusion.

North Dakota Century Code dictates that every birth occurring in the state must be registered within ten days of delivery; and as stated in the North Dakota Century Code 14-15-07 the petition for adoption must be executed at any time *after* the birth of a child. This time-intensive process ends up taking approximately six months to complete, thereby making the identification of adoption cases almost impossible to identify in the birth file at the time of the ND PRAMS sampling. Additionally, adoptions in North Dakota are processed by the Department of Human Services, which is a separate entity from the Department of Health’s, Division of Vital Records, which can cause delays in the reporting of adoptions.

<http://www.legis.nd.gov/cencode/t14c15.pdf>

1. ***Surrogate Births.*** Current medical technology has made it possible for couples to have a baby through surrogacy. Unlike the process of adoption, no legal consensus has been reached about surrogate motherhood in the United States. As a result, the sampling of an infant delivered by a surrogate carrier (or gestational carrier) can be a sensitive issue. Under certain circumstances the intended mother (the woman who will raise the child) is named on the birth certificate. Since the majority of survey questions involve the time period prior to and during the pregnancy, the intended mother is not qualified to respond to the PRAMS survey. For this reason, any births that can be identified as such should be excluded from the sample.

If a surrogate birth is not identified for exclusion prior to drawing the sample but is discovered during the data collection period, one of two situations may occur. In the first case, if the name of the intended mother is provided on the birth certificate, this mother should be dropped from further follow-up for the reasons given above (using the "Drop Mom" feature in the PRAMS software system). If, however, the name of the surrogate carrier is provided on the birth certificate, PRAMS staff should attempt to contact this woman and encourage her to participate. This woman will be able to answer the questions regarding her experiences prior to and during the pregnancy. The survey design will also allow these women to skip those questions pertaining to the child's early infancy.

### Protocol Development Task

Explain how surrogate births are processed in your state and explain if and how they are identifiable for exclusion.

North Dakota Century Code 14-18-01 states that a child born to a gestational carrier is the child of the intended parents for all purposes. However, surrogacy agreements where a surrogate contributes the egg; and/or where the child’s father is the surrogate’s husband are considered void and unenforceable. Given that a birth certificate has to be filed within 10 days of a birth in North Dakota, identifying these cases for exclusion is not possible at the time of sampling, since a birth certificate is already likely to be filed in the name of the child’s intended parents.

<http://www.legis.nd.gov/cencode/t14c18.pdf>

1. ***Additional State-Required Exclusions.***

### Protocol Development Task

Your state may have additional exclusions that are required by law or because of the nature of your vital records processing. If you have any state-required exclusions, list them here beginning with 4.2a_viii_. Define what the exclusions will be and estimate their expected numbers.

No other exclusions are required by state law. North Dakota resident births that will participate in the SD Tribal PRAMS will be included as an additional strata to the ND PRAMS, upon completion.

***4.2b*** ***Assessment of the Exclusions on the Generalizability of the Sampling Frame.*** It is important to understand the effects of exclusions from the frame on potential generalizability of the results to the total birth population. We anticipate that the magnitude of the exclusions listed above will be minimal with the possible exception of out-of-state births to residents. The tables below show sample calculations of one state's exclusions by stratification variable

***Table 4.*2ai: North Dakota 2015 Resident Births**

| **Stratifi-**  **cation Variable** | **Number Born Inside State (to Table 4.2b_iii_)** | **Row / Col Pct**  **Inside State** | **Number Born Outside State** | **Row / Col Pct Outside State** | **Number of**  **Total Resident Births** | **Row / Col Pct Total Resident Births** |
| --- | --- | --- | --- | --- | --- | --- |
| **Non-American Indian** | 9944 | 97.7/90.3 | 230 | 2.3/91.2 | 10174 | 100/90.3 |
| ****American Indian not in Tribal Sample** | 930 | 97.7/8.5 | 22 | 2.3/8.8 | 952 | 100 /8.4 |
| **ND Tribal Sample** | 137 | 100/1.2 | N/A | N/A | 137 | 100/1.2 |
| **GRAND TOTAL** | 11011 | 97.7/100 | 252 | 2.2/100 | 11263 | 100/100 |

*Total number of ND resident births was 11265, 2 records to out of state births (254) were missing maternal race information. Grand Totals include ND Resident Births and SD Tribal Resident births

**American Indian Race includes women reporting both American Indian and White Race

Source: *North Dakota, Division of Vital Records, 2015*

**** ND American Indian not in Tribal Sample is the total number of ND AI resident births – ND AI residents in Tribal Sample (1067-137)*

****Total AI Tribal Sample is the number of AI residing in SDT Counties in ND and SD, occurring in ND*

**Table 4.2bi: North Dakota 2015 Resident Births with Complete Information**

| **Stratifi-**  **cation Variable** | **Number With Complete Information (to Table 4.2bv)** | **Row / Col Pct Complete Information** | **Number With Missing Information** | **Row / Col Pct**  **Missing Information** | **Total (from Table 4.2bi)** | **Row / Col Pct**  **Total** |
| --- | --- | --- | --- | --- | --- | --- |
| **Non-American Indian** | 9942 | 99.9/90.3 | 2 | .002/100 | 9944 | 100/90.3 |
| ****American Indian** | 930 | 100/8.5 | 0 | 0/0 | 930 | 100 / 9.7 |
| **ND Tribal Sample** | 137 | 100/1.2 | N/A | N/A | 137 | 100/1.2 |
| **GRAND**  **TOTAL** | 11011 | 97.7/100 | 2 | 2.2/100 | 11011 | 100/100 |

**Table 4.2bii: Multiple Deliveries with Complete Information**

| **Stratifi-**  **cation Variable** | **Total Births (from Table 4.2b_iii_)** | **Singleton Births/**  **Mothers** | **Number of Births With Plurality = 2** | **Mothers Giving Birth to Twins** | **Number of Births With Plurality =3** | **Mothers Giving Birth to Triplets** | **Number of Births With Plurality>=4** | **Total PRAMS-eligible Population of Mothers (to Table 4.3.c)** |
| --- | --- | --- | --- | --- | --- | --- | --- | --- |
| **Non-American Indian** | 9944 | 9630 | *311 | 156 | 3 | 1 | 0 | 9787 |
| **American Indian not Tribal** | 1067 | 1045 | 22 | 11 | 0 | 0 | 0 | **919 |
| **GRAND TOTAL** | 11,011 | 10675 | 33 | 167 | 3 | 1 | 0 | 10717 |

### Protocol Development Task

1. After selecting your stratification variables **(Section 4.3b)**, return to this task box. Create tables identical with the examples above for *each* of your state's exclusions numbered 4.2a_i_, 4.2a_iii_, 4.2a_v_ - 4.2a_vii_ and for any additional exclusions identified beginning with 4.2a_viii_.

The state exclusions relevant to ND PRAMS are presented in the tables above. No exclusions are made based on adoption, surrogacy or delayed birth certificates.

1. For residents giving birth out of state (4.2b_i_), records with missing information (4.2b_iii_), adoptions (4.2b_vi_), and surrogate births (4.2b_vii_), describe the differences between the strata for the excluded and the included (the sample frame) records for the exclusions that are of substantial magnitude that they may affect the population. What proportion do the excluded records represent of the total birth population of interest?

Approximately 2 percent of deliveries, both in the American Indian (2.0) and non-American Indian (2.3) births were born outside the state. A majority of the out of state births occurred in Minnesota and South Dakota, which is expected given the border hospitals as well as tribal reservations that cross into both MN and SD.

1. If there are differences in the sampling frame regarding stratification characteristics caused by an exclusion as compared with the true birth population of the state, then the sample will not be an accurate reflection of the population it is meant to represent. For residents giving birth out of state (4.2b_i_ ), records with missing information (4.2b_iii_ ), adoptions (4.2b_vi_ ), and surrogate births (4.2b_vii_ ), explain how the generalizability of your estimates will be influenced by these differences.

The proportions of out of state deliveries in the strata do not differ significantly enough to affect the generalizability of statewide data. Adoptions and surrogate births are not identifiable in the ND birth file without linkage to the ND DHS adoption files, therefore they will not be excluded in the sampling frame.

1. Place your newly developed tables describing each exclusion (4.2a_i_, 4.2a_iii_, 4.2a_v_-4.2a_vii_) following the description of that exclusion.

Tables with the relevant exclusions are shown above. No additional exclusions or changes.

## Sampling Plan

***4.3a*** ***Why Use Stratified Sampling?*** For PRAMS surveillance, there is often a particular interest from a public health perspective in certain subpopulations. These subpopulations may not represent a large portion of a state's overall population. To make inferences about specific subpopulations and make comparisons among several subpopulations, infants in those subpopulations (commonly called *strata*) will need to be oversampled (i.e., sampled at a higher rate than other subpopulations).

The main advantage of *stratified sampling* is that it permits separate estimates of subgroups of interest and permits comparisons across these subgroups.

An alternative to a stratified sample is a *proportional sample*. With proportional sampling, specific subpopulations are represented in the sample in the same proportion as they are represented in the actual population. Thus, if 3% of a state's births are low birthweight, about 3% of the sample would be expected to be low birthweight. In the context of PRAMS, the main advantages of proportional sampling over stratified sampling are that weights are not always required, which makes the analysis of such data simpler, and statewide prevalence estimates will be more accurate and have smaller variance.

For PRAMS, however, the ability to make inferences about subgroups of high public health interest and to make comparisons across the groups is important. This need outweighs the difficulties of computing and using sampling weights. Therefore, PRAMS incorporates a stratified sampling scheme.

***4.3b*** ***Stratification Scheme.*** The sampling plan is designed so that inferences about prevalence rates for maternal behaviors can be estimated with sufficient precision both at the state level and within selected strata. The choice of stratification variables is limited to the information available on birth certificate records. For reasons of data quality and the efficiency of estimates, this choice of variables is further limited to information reported with high accuracy.

### Protocol Development Task

Your state may choose up to two stratification variables from the following list:

Birthweight Maternal Age

Maternal Race and Ethnicity Geographic Area

Maternal Education Medicaid Status

To limit annual samples to a manageable size, each stratification variable can have from two to four levels, but the total number of strata cannot exceed six. For example, you may choose to have one stratification variable with three levels; two stratification variables with two levels each; or two stratification variables, one with three levels and one with two levels. In addition, separate strata may be created for unknowns.

States are strongly encouraged to choose a single stratification variable in their first year of data collection to help keep the sample manageable. The sampling plan can be modified in the second year of operations to stratify by an additional variable, depending on the state's success in contacting hard-to-reach mothers.

Any stratification variable you define must be included in birth certificate sample and frame files if not part of the core variables. These variables must further be added to the state-specific portion of the file.

Provide the following information where relevant in the preceding section:

1. Description and detailed rationale for the stratification scheme selected.

North Dakota will be stratifying by maternal race. This is due to the significant disparities in maternal and infant outcomes between American Indians and other races in the state. Due to the small numbers in the other races and comparable outcomes, ND will be combining white and the other races as the comparison group. Proportion of 2015 births for Black, Hispanic, Asian; and Other/Unknown were: 4.5% (547); 5.1 (557); 2.4 (266) and 3.4 (376).

2. Definitions of all strata.

Maternal Race: American Indian; and American Indian who also report as biracial with white race; compared to the Non-American Indian maternal race

***4.3c*** ***Determining Sample Size.*** Required sample sizes for PRAMS are determined in relation to the given proportion that is being estimated, at a given level of precision, and with a given level of statistical confidence. For specified levels of precision and confidence, the sample size required is at its maximum when the advance estimate (the number used in sample size calculations) of the proportion being estimated equals 0.50. PRAMS data are used in estimating proportions for risk factors that range from common (such as delivery paid for by Medicaid) to rare (such as drinking alcohol during pregnancy). Using 0.50 in sample size calculations leads to the largest sample sizes for a specified level of precision and confidence, whatever the true population proportions are for the various risk factors.

Based on the stratification measures found above, a sample size of about 400 (n = 400) is necessary in each stratum to estimate a dichotomous variable with reasonable precision and 95% confidence, assuming an infinitely large population size (N). The assumption of an infinitely large population will be violated in the oversampled strata. In any stratum where the desired sample size of 400 comprises more than 5% to 10% of the population, it is appropriate to apply the Finite Population Correction (FPC). The FPC will reduce the desired sample sizes in such cases without compromising the precision of the estimates.

The formula for FPC is:

adjusted size = n / (1 + (n/N)),

where n = desired sample size,

N = population size.

Mothers in some strata may be more difficult to contact than mothers in other strata. Thus, actual stratum sample sizes must be larger than theoretically needed to achieve a given level of statistical power. Based on the estimated stratum-specific response rates, the stratum-specific sample sizes will be inflated to ensure an adequate number of responses for analysis.

This practice, as noted by Don Dillman, author of *Mail and telephone surveys: The Total Design Method^[[1]](#footnote-1)^* and a consultant to PRAMS during its initial development, amounts to substituting "an available respondent for one that cannot be contacted." Dillman quotes the statistician W. Edward Deming, who said that "Substitution does not help; it is only equivalent to building up the size of the initial sample, leaving the bias of nonresponse undiminished." To assess validity, one cannot compensate for a low response rate by inflating the numbers in the strata. Increasing the sample size does reduce the random component of error in estimates obtained from PRAMS. Nonrandom or systematic error from response bias can best be reduced by improving response rates.

When calculations lead to a high sampling fraction (greater than half) and the cost is not prohibitive, common survey practice is to select all the records in a stratum. Small strata are sometimes volatile, and creating a take-all stratum guards against the shortfall in responses that an unexpected decline in births could cause. If such a decline is unlikely, however, 100% sampling is usually unwarranted. Increasing the sample beyond the calculated initial size in small strata brings little gain in precision for the expense incurred. Given typical PRAMS states’ budgets and standards for precision, 100% sampling is out of the question if the stratum population size exceeds 571 (minimum sample size of 400 inflated by reasonably attainable response rate of 70%). Even if the stratum population size is below 571, 100% sampling is inadvisable because it is possible to get precise estimates without using 100% of the sample.

In the context of a state-wide, population-based surveillance system like PRAMS, it may not be reasonable to select as strata demographic groups comprising less than 2% of the population or fewer than 500 women. States must balance competing priorities and give careful consideration to precluded opportunities when choosing groups to oversample.

To reduce nonresponse bias, one of the components of the analysis weights (See **Chapter 6, Section 6.8 and Appendix B** for a discussion of analysis weights) adjusts for nonresponse patterns because response rates vary among strata. However, weights may not adequately compensate for low response rates. The nonresponse weight assumes that the average of the answers of the respondents within a particular stratum and response category under consideration is the same as the average of the answers of the nonrespondents in that stratum and response category. Although the assumption seems reasonable for strata with high response rates, it becomes increasingly implausible for strata with lower response rates. For strata with response rates below 50%, this assumption is unjustified.

### Protocol Development Task

1. For each stratification variable, provide the following information where relevant:

a. Determine the estimated annual population and proposed annual sample size for each stratum. Provide the rationale for all sample sizes.

Estimated annual population is based on 2015 North Dakota Vital Records data. Estimated unadjusted sample size is 400 per strata per CDC protocol. Finite population correction factor was employed in the Non-American Indian group since the estimated unadjusted sample size is 14.6 percent.

b. Determine the anticipated response rates and provide rationale.

Anticipated response rates: Estimated response rates for American Indian women are based on the South Dakota 2014 PRAMS-like survey, which followed the CDC protocol with a few modifications (no 3^rd^ mailing, online version, texting).

c. Determine the level of precision for the anticipated analyses.

Level of precision: The unadjusted sample size of 400, based on a risk factor proportion of 0.50 with a desired precision of +/- 0.05.

NOTE: Response rates are typically influenced by two main factors: the study population and the methodology employed. If your state has conducted other research studies on similar populations, you may be able to use response rates from those studies to estimate response rates for PRAMS. However, if the study population is different from the PRAMS target population, results from these studies may not be useful predictors for PRAMS. The mail/telephone methodology used by PRAMS typically elicits higher response rates than those encountered with most mail-out surveys. Response rate results from studies that used other modes of data collection may not be comparable. Based on the experiences of states conducting PRAMS, the expected response rates average between 60% and 70% for high-risk strata and between 65% and 80% for low-risk strata, and the annual samples will range from 1,600 to 2,500 infants.

d. See table 4.3c below

The following table illustrates this state's figures and provides an example of how your state can choose to present your figures.

**Example Table 4.3.c**

**Calculation of Annual Sample Size**

| Stratum | **PRAMS Population Size**  **(from Table**  **4.2b_v_, and to Table 4.4b)** | **Estimated Annual Unadjusted Sample Size** | **FPC**  **Corrected Sample**  **Size^#^** | **Estimated Response Rates** | **Estimated**  **Annual**  **Sample Size**  **Adjusted for Non-response* (to Table 4.4b)** |
| --- | --- | --- | --- | --- | --- |
| **Non-American Indian** | 9787 | 400† | 384 | 60% | 670 |
| **American Indian** | 919 | (ND AI Census Sample) | 786 | **49% | 919 |
| **Total** | 10717 | 1330 | 1168 |  | 1559 |

^# Finite Population Correction (FPC)^

* Adjusted sample sizes calculated by dividing unadjusted sample sizes (or FPC when applicable) by response rates (as proportions).

† Sample size estimates calculated assuming a risk factor proportion of 0.50, desired precision of +/-0.05, and 95% confidence interval. Calculations use formula from Levy, P.S. and S.L. Lemeshow, *Sampling for Health Professionals*, Belmont, California: Lifetime Learning Publication, 1980.

**Estimated response rate of American Indian mothers is based on the SD PRAMS-like survey.

Source of birth data (column one): *North Dakota Division of Vital Records, 2015*

***4.3d*** ***Evaluation of Sample Size.*** Because birth distributions and response rates can change over time, CDC has developed procedures for evaluating the sampling scheme on a regular basis and making modifications if necessary. For further information regarding the evaluation of sample size procedures see **Section 11.1a.**

## Selection of Sample

Producing a sample of mothers has two steps, constructing the frame and drawing the sample.

***4.4a*** ***Preparation of the Frame.*** North Dakota's vital records birth certificate file serves as the source of the sampling frame representing live-born infants. Records not yet processed are not available for inclusion on the frame. Records identified as exclusions as listed in **Section 4.2** are excluded as part of the construction of the frame. No infant should be included in the sampling frame twice. The sampling frame will be routinely checked for duplicate records to eliminate the possibility of an infant being included twice.

### Protocol Development Task

Describe in detail the birth registration process including time frames for this process. If there are separate procedures for electronic birth certificates, describe them as well. Specify the proportion of certificates that are obtained by each method that will be available for sampling during a range of time periods after birth. It may be useful to display this information in a flowchart (see example below). Be sure to describe when and how the identifying number (sometimes referred to as the state file number or birth certificate number) is added to the records. Describe exactly where the PRAMS sampling procedures take place in the registration process.

The specifics of the way your state processes records may vary, but your protocol should include comparable detail as shown in the example below.

North Dakota Century Code requires registration of all births within ten days of the date of birth. The NDDoH, Division of Vital Records (DVR), maintains an average of eight days between a birth and registration of the birth record.

This process of Birth Registration is a combined process using the Electronic Vital Event Registration System (EVERS) – a web based tool provided by the DVR. The DVR provides a Parent’s Worksheet and a Certifier’s Worksheet to birthing facilities to serve as data collection tools. The data entry of information collected takes place at the birthing facility. This is a two-person process to insure that no one person can create an identity. A data entry clerk enters all information from both worksheets and submits the record for review by a submitting clerk. Upon review of all data items, the record is submitted to the DVR. When a birth record is submitted to the DVR, the record is considered registered and a certificate number is assigned. If a record requires voluntary acknowledgment of paternity, such as when a child is born out of wedlock, the form is required before the birth is registered. When birth records are registered, the data from this birth records are immediately available for use in several different applications.

The timeliness of the DVR in the birth and death registration process in North Dakota will provide timely monthly vital records data to ensure that mothers are sampled within the recommended time frame of two to four months.

The PRAMS sample will be drawn once the records have been received, coded, keyed, and edited.

As part of the process of developing the sampling frame, the file of birth certificate records is divided into strata, from each of which a sample is drawn. Preparing the frame also includes deleting the records for all but one infant in each multiple birth. One easy manner to accomplish this selection involves basing the selection on birth order and date of birth (DOB). For example, for twins, choose the first-born if the DOB lies between the first and fifteenth of the month; otherwise choose the second-born. Similarly, the month can be broken down into three segments for triplets. This selection process is used only if all the deliveries resulted in a live birth. If only one twin was born alive, it is treated as a singleton birth for sampling purposes.

### Protocol Development Task

1. Describe the preparation of the records before sampling. The procedures for preparing the records before sampling will include development of the stratum-specific sampling frames, identifying and eliminating exclusions, and dealing with records for multiple births.

The Deputy State Registrar reviews all birth certificate data on a monthly basis. She will then download a monthly birth file for sampling and securely share it with the PRAMS Program Manager to run the sampling program, shown below, and draw the sample.

1. The following two flow charts show signal events in the processing of birth certificate records and preparation of the frame. The first example shown is of the process utilized for a paper certification system. Please adapt the flow chart that follows to your state-specific situation. If you process your birth certificates via EBC or use a combination of both, include a flowchart for each.

| Flow of Vital Records  Birth  ⭣  Birth Certificate Completed in the ND DVR  ⭣  State Registrar  ⭣  Data Entry at Office of Vital Records  ⭣  Birth Database in the ND PRAMS Office  ⭣  Copy of Birth Database Placed on ITD Secured Server  ⭣  Sampling Frame ran by ND PRAMS Staff |
| --- |

| FLOWCHART OF COMPUTER PROCESS FOR SELECTING **NORTH DAKOTA STATE PRAMS** SAMPLING FRAME AND SAMPLE (for states using mail/telephone surveillance) | | | | |
| --- | --- | --- | --- | --- |
|  | | | | READ **RESIDENT BIRTH RECORD |
|  |  |  |  | 🡓 |
| EXCLUDE | 🡐 | YES | 🡐 | DID BIRTH OCCUR IN ANOTHER STATE? |
|  | | | | 🡓 NO |
| EXCLUDE | 🡐 | YES | 🡐 | WAS INFANT ADOPTED OR DELIVERED BY A SURROGATE MOTHER? |
|  | | | | 🡓 NO |
| EXCLUDE | 🡐 | YES | 🡐 | DID BIRTH OCCUR MORE THAN 6 MONTHS BEFORE SAMPLING DATE? |
|  | | | | 🡓 NO |
| EXCLUDE FROM THIS SAMPLING FRAME AND INCLUDE IN NEXT ELIGIBLE SAMPLING FRAME | 🡐 | YES | 🡐 | DID BIRTH OCCUR LESS THAN 2 MONTHS BEFORE SAMPLING DATE? |
|  | | | | 🡓 NO |
| RANDOMLY SELECT ONE FOR INCLUSION IN SAMPLING FRAME | 🡐 | YES | 🡐 | IS THIS BIRTH IN A MULTIPLE GESTATION? |
|  | | | | 🡓 NO |
| EXCLUDE | 🡐 | YES | 🡐 | IS MOTHER AMERICAN INDIAN WHO RESIDES IN  Tribal-PRAMS COUNTY? ^ |
|  | | | | 🡓 NO |
|  | | | | 1. ASSIGN TO ONE OF THE SAMPLING STRATA  2. EXTRACT BIRTH CERTIFICATE INFORMATION |
| NOT IN SAMPLE | 🡐 | NO | 🡐 | SELECTED FOR SAMPLE? |
|  | | | | 🡓 YES |
|  |  |  |  | EXTRACT INFORMATION FROM THE SAMPLE FILE TO PRODUCE:  1. INITIAL CONTACT LETTERS  2. FOLLOW-UP LETTER  3. MAILING LABELS |

*Resident Birth Files contains birth records of North Dakota Residents Only – i.e. births to North Dakota residents both in state and out of state

| FLOWCHART OF COMPUTER PROCESS FOR SELECTING **NORTH/SOUTH DAKOTA’S TRIBAL PRAMS** SAMPLING FRAME AND SAMPLE (for states using mail/telephone surveillance) | | | | |
| --- | --- | --- | --- | --- |
|  | | | | READ ND *OCCURENT BIRTH RECORD |
|  |  |  |  | 🡓 |
| EXCLUDE | 🡐 | YES | 🡐 | DID BIRTH OCCUR IN ANOTHER STATE? |
|  | | | | 🡓 NO |
| EXCLUDE | 🡐 | YES | 🡐 | WAS INFANT ADOPTED OR DELIVERED BY A SURROGATE MOTHER? |
|  | | | | 🡓 NO |
| EXCLUDE | 🡐 | YES | 🡐 | DID BIRTH OCCUR MORE THAN 6 MONTHS BEFORE SAMPLING DATE? |
|  | | | | 🡓 NO |
| EXCLUDE FROM THIS SAMPLING FRAME AND INCLUDE IN NEXT ELIGIBLE SAMPLING FRAME | 🡐 | YES | 🡐 | DID BIRTH OCCUR LESS THAN 2 MONTHS BEFORE SAMPLING DATE? |
|  | | | | 🡓 NO |
| RANDOMLY SELECT ONE FOR INCLUSION IN SAMPLING FRAME | 🡐 | YES | 🡐 | IS THIS BIRTH IN A MULTIPLE GESTATION? |
|  | | | | 🡓 NO |
| **Tribal-PRAMS**  **ND Occurrent Births Residing in Tribal Counties**  **File Sent to GPTCHB** | 🡐 | YES | 🡐 | IS MOTHER AMERICAN INDIAN WHO RESIDES IN  Tribal-PRAMS COUNTY? ^ |

*ND Occurrent Birth File contains all the births occurring in North Dakota i.e. residents and non-residents delivering in North Dakota

**Tribal Sample will include Births occurring in North Dakota to residents of: **Standing Rock Sioux Tribe**:  South Dakota counties of Campbell, Corson, Perkins, & Walworth and North Dakota counties of Adams, Emmons, Grant, Morton & Sioux. **Crow Creek Sioux Tribe**:  South Dakota counties of Brule, Buffalo, Hand, Hughes & Hyde. **Flandreau Santee Sioux Tribe**:  Moody County, South Dakota. **Sisseton-Wahpeton Oyate (SWO)**:  South Dakota counties of Codington, Day, Grant, Marshall, & Roberts; North Dakota counties of Richland & Sargent

***4.4b Selecting the Sample.*** The selection procedures must satisfy the probability requirements of the sample. The sample is chosen so that, within each stratum, each record has an equal probability of being selected. Based on these probabilities, weights can be determined for statewide estimates.

Systematic sampling within each stratum is used. The probability of being selected, commonly referred to as the sampling fraction (*1/f*) for a given stratum, is based on the estimated size of the sampling frame for the stratum and the desired sample size for the stratum. *f* is computed by dividing the population size by the sample size (i.e., *f* = N/n). A random number between 1 and *f* is chosen, and that record, as well as every *f-th* record thereafter, is selected for the sample.

### Protocol Development Task

Compute sampling fractions for each stratum, and display them in table form, as illustrated in the following example.

See below

**Table 4.4b**

PRAMS Sampling Fractions and Estimated Sample Sizes by Stratum

**(Based on 2015 South Dakota Vital Records Data)**

| Stratum | **PRAMS Population Size (from Table 4.3c)** | **Estimated Adjusted Sample Size (from Table 4.3c)** | **f = N/n** | **f = N/n expressed as common fraction** | **Operational Sample Size, Annual** | **Operational Sample Size, Monthly** |
| --- | --- | --- | --- | --- | --- | --- |
| **Non-American Indian** | 9787 | 640 | 14.6 | 15 | 640 | 53 |
| **American Indian** | 919 | 919 | 1 | 1 | 919 | 77 |
| **Total** |  | 1559 |  |  | 1559 | 130 |

*Tribal Sample includes Births occurring in North Dakota to residents of: **Standing Rock Sioux Tribe**:  South Dakota counties of Campbell, Corson, Perkins, & Walworth and North Dakota counties of Adams, Emmons, Grant, Morton & Sioux. **Crow Creek Sioux Tribe**:  South Dakota counties of Brule, Buffalo, Hand, Hughes & Hyde. **Flandreau Santee Sioux Tribe**:  Moody County, South Dakota. **Sisseton-Wahpeton Oyate (SWO)**:  South Dakota counties of Codington, Day, Grant, Marshall, & Roberts; North Dakota counties of Richland & Sargent

**Example Table 4.4b**

PRAMS Sampling Fractions and Estimated Sample Sizes by Stratum

**(Based on 2013 Vital Records Data)**

## Frequency and Timing of Sampling

The timetable for sampling is expressed in terms of actual calendar time and in terms of the approximate length of time after delivery. The particular day chosen to sample may depend on vital records processing. The schedule for drawing the sample permits births to be sampled within two to six months after delivery. However, the ideal sampling period is two to four months after delivery. To collect information about factors that occur in early infancy, mothers are contacted no earlier than two months after delivery. Sampling on a monthly basis ensures that mothers are contacted in a timely manner and that there is a balanced workload for PRAMS staff.

### Protocol Development Task

Describe when the sampling frame will be constructed and when the sample will be drawn. Estimate the number of birth certificates that will be missed because of delayed registration with the sampling frame you have established.

Complete this statement:

A sample will be drawn on the 20th day (every third Monday) of each month. This will make the records 2 to 6 months old.

1. Dillman DA (1978). *Mail and telephone surveys: the total design method*. New York: Wiley-Interscience [↑](#footnote-ref-1)
