# Supplementary material for: Impact of Breastfeeding Barriers on Racial/Ethnic Disparities in Breastfeeding Outcomes in North Dakota
Source: J Racial Ethn Health Disparities. 2024 Feb 23;12(2):1063–72. doi: 10.1007/s40615-024-01943-z (PMC11913940; doi:10.1007/s40615-024-01943-z)
Supplement: Supplementary file 3 — Supplementary file3 (DOCX 18 KB) [file 40615_2024_1943_MOESM3_ESM.docx]

| **Outcomes** | |  |
| --- | --- | --- |
|  | Breastfeeding status at 2 months (yes/no) | |
|  | Breastfeeding status at 4 months (yes/no) | |
|  | Time to cessation of breastfeeding in weeks (continuous) | |
| **Breastfeeding Barriers** | |  |
|  | My baby had difficulty latching or nursing | |
|  | Breast milk alone did not satisfy my baby | |
|  | I thought my baby was not gaining enough weight | |
|  | My nipples were sore, cracked, or bleeding or it was too painful | |
|  | I thought I was not producing enough milk, or my milk dried up | |
|  | I had too many other household duties | |
|  | I felt it was the right time to stop breastfeeding | |
|  | I got sick or I had to stop for medical reasons | |
|  | I went back to work | |
|  | I went back to school | |
|  | My partner did not support breastfeeding | |
|  | My baby was jaundiced (yellowing of the skin or whites of the eyes) | |
|  | Other | |
| **Race of Birthing Parent** | |  |
|  | American Indian/Alaska Native (AIAN alone or biracial AIAN-White) | |
|  | White (White alone) | |
|  | Other Racial Identities (other race/ethnicity; includes Black, Asian, Hispanic [all races], and other/unknown) | |
| **Covariates** | |  |
|  | Parental age (younger than 35, and 35 and older) | |
|  | Prenatal insurance type (Medicaid, other, and none) | |
|  | Prenatal care adequacy (Kotelchuck index: Inadequate, Intermediate, Adequate, Adequate Plus) | |
|  | Chronic illness (has at least one chronic illness, or no chronic illness) | |
|  | Substance use (used tobacco or alcohol in the past 2 years, or did not use tobacco or alcohol in the past two years) | |
|  | Weight classification (overweight or not overweight) | |
|  | Postpartum depression (Always/Often, Sometimes, or Rarely/Never) | |
|  | History of depression (present, or not present) | |
|  | Education (high school or more, or did not finish high school) | |
|  | Income (less than $40,000 annually, or $40,000 or higher annually) | |
|  | Use of WIC program during pregnancy (yes, or no), | |
|  | Adverse childhood experiences (Ace score: <2, or ≥2) | |
|  | Pregnancy intention (later, sooner, at this time, not wanted, not sure)  Infant sleep position (back, non-back)  Infant sleeping alone (Always/Often, Sometimes/Rarely/Never) | |

**Online Resource 3. Brief description of all variables included.**
