# Supplementary material for: Impact of Breastfeeding Barriers on Racial/Ethnic Disparities in Breastfeeding Outcomes in North Dakota
Source: J Racial Ethn Health Disparities. 2024 Feb 23;12(2):1063–72. doi: 10.1007/s40615-024-01943-z (PMC11913940; doi:10.1007/s40615-024-01943-z)
Supplement: Supplementary file 4 — Supplementary file4 (DOCX 18 KB) [file 40615_2024_1943_MOESM4_ESM.docx]

**Online Resource 4. Sensitivity analysis results with infant sleep variables added to logistic regression models estimating odds of breastfeeding duration by race/ethnicity.**

|  | **Breastfeeding at Two Months**  **(OR, 95% CI)** | | | **Breastfeeding at Four Months**  **(OR, 95% CI)** | | | |
| --- | --- | --- | --- | --- | --- | --- | --- |
|  | **Unadjusted^1^** | **Adjusted for covariates^2^** | **Adjusted for covariates and other barriers^3^** | **Unadjusted^1^** | **Adjusted for covariates^2^** | **Adjusted for covariates and other barriers^3^** |  |
| American Indian |  |  |  |  |  |  |  |
| vs. White | 0.39 (0.32,0.48) | \| 0.55 (0.37, 0.82) \| \| --- \| | \| 0.83 (0.44, 1.57) \| \| --- \| | 0.33 (0.27,0.39) | \| 0.48 (0.33, 0.71) \| \| --- \| | \| 0.89 (0.27, 2.90) \| \| --- \| |  |
| Other vs. White | 1.08 (0.74,1.58) | \| 0.95 (0.48, 1.88) \| \| --- \| | \| 2.01 (0.78, 5.14) \| \| --- \| | 0.93 (0.67,1.29) | \| 0.85 (0.45, 1.59) \| \| --- \| | \| 2.99 (0.95, 9.46) \| \| --- \| |  |

^1^Crude association between explanatory and outcome variable

^2^Adjusted for age, income, education, insurance used for prenatal care, use of WIC program during pregnancy,

Kotelchuck index of prenatal care adequacy, ACE score, history of depression, history of chronic disease,

substance use, overweight status, infant sleep, and postpartum depression

^3^Adjusted for covariates named above, plus all breastfeeding barriers

Shaded cells indicate statistically significant (p<0.05)
