# Supplementary material for: Impact of Breastfeeding Barriers on Racial/Ethnic Disparities in Breastfeeding Outcomes in North Dakota
Source: J Racial Ethn Health Disparities. 2024 Feb 23;12(2):1063–72. doi: 10.1007/s40615-024-01943-z (PMC11913940; doi:10.1007/s40615-024-01943-z)
Supplement: Supplementary file 5 — Supplementary file5 (DOCX 17 KB) [file 40615_2024_1943_MOESM5_ESM.docx]

**Online Resource 5. Sensitivity analysis results with infant sleep variables added to hazard models estimating breastfeeding cessation by race/ethnicity.**

|  | **Hazard for cessation of breastfeeding**  **HR (95% CI)** | | |
| --- | --- | --- | --- |
|  | **Unadjusted^1^** | **Adjusted for covariates^2^** | **Adjusted for covariates and other barriers^3^** |
| American Indian vs. White | 0.97 (0.85,1.10) | 0.84 (0.66,1.06) | 0.78 (0.6,1.02) |
| Other vs. White | 0.78 (0.63,0.97)* | 0.76 (0.53,1.10) | 0.73 (0.50, 1.06) |

^1^Crude association between explanatory and outcome variable

^2^Adjusted for age, income, education, insurance used for prenatal care, use of WIC program during pregnancy,

Kotelchuck index of prenatal care adequacy, ACE score, history of depression, history of chronic disease,

substance use, overweight status, infant sleep and postpartum depression

^3^Adjusted for covariates named above, plus all breastfeeding barriers
