# Supplementary material for: Impact of Breastfeeding Barriers on Racial/Ethnic Disparities in Breastfeeding Outcomes in North Dakota
Source: J Racial Ethn Health Disparities. 2024 Feb 23;12(2):1063–72. doi: 10.1007/s40615-024-01943-z (PMC11913940; doi:10.1007/s40615-024-01943-z)

**Online Resource 6.** Fully adjusted survival Curves for time to cessation of breastfeeding by race, with infant sleep variables included in model.


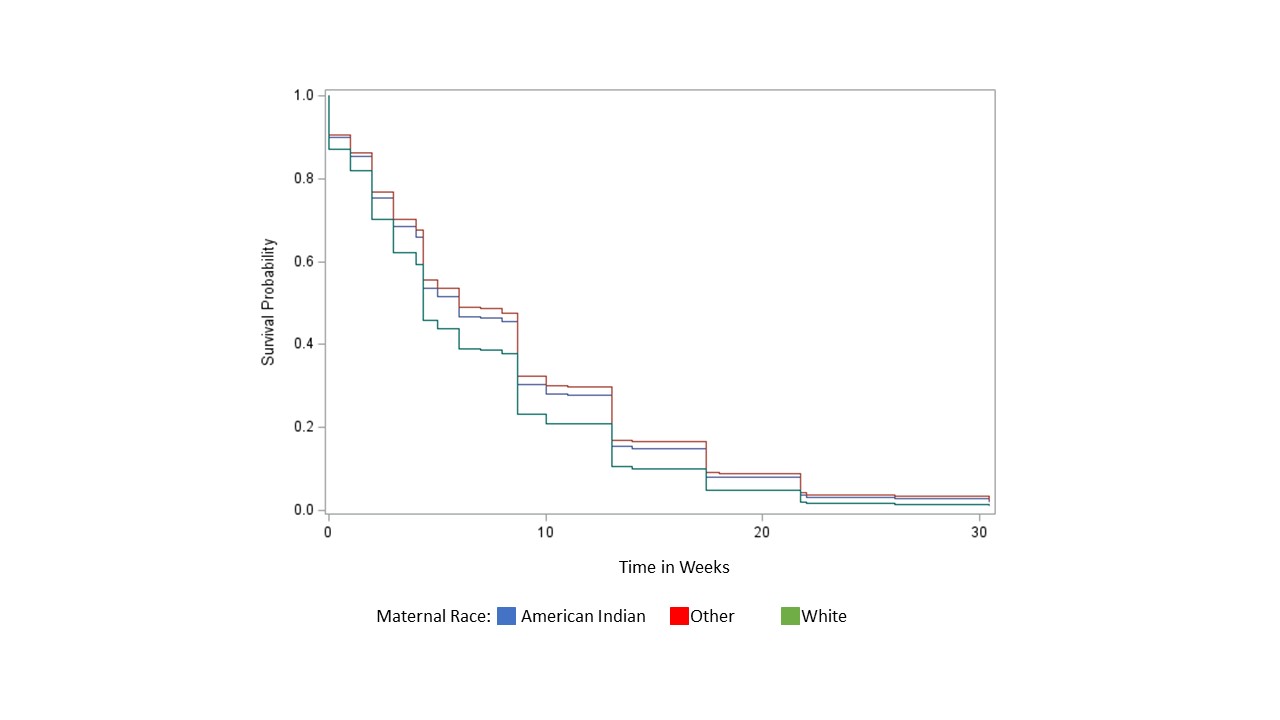

Supplement: Supplementary file 6 — Supplementary file6 (DOCX 55 KB) [file 40615_2024_1943_MOESM6_ESM.docx]
